# Target identification of small molecules: an overview of the current applications in drug discovery

**DOI:** 10.1186/s12896-023-00815-4

**Published:** 2023-10-10

**Authors:** Yasser Tabana, Dinesh Babu, Richard Fahlman, Arno G. Siraki, Khaled Barakat

**Affiliations:** 1 Faculty of Pharmacy and Pharmaceutical Sciences, University of Alberta, Edmonton, AB Canada; 2 Department of Biochemistry, University of Alberta, Edmonton, AB Canada; 3 Li Ka Shing Applied Virology Institute, University of Alberta, Edmonton, AB T6G 2E1 Canada

**Keywords:** Small molecule, Target identification, Affinity-based pull-down, Label-free

## Abstract

Target identification is an essential part of the drug discovery and development process, and its efficacy plays a crucial role in the success of any given therapy. Although protein target identification research can be challenging, two main approaches can help researchers make significant discoveries: affinity-based pull-down and label-free methods. Affinity-based pull-down methods use small molecules conjugated with tags to selectively isolate target proteins, while label-free methods utilize small molecules in their natural state to identify targets. Target identification strategy selection is essential to the success of any drug discovery process and must be carefully considered when determining how to best pursue a specific project. This paper provides an overview of the current target identification approaches in drug discovery related to experimental biological assays, focusing primarily on affinity-based pull-down and label-free approaches, and discusses their main limitations and advantages.

## Introduction

Target identification is a crucial stage in the discovery and development of new drugs since it enables researchers to understand the mode of action of enigmatic drugs [1]. For this reason, much of the progress made in drug discovery and development over the past two centuries can be attributed to advances in target identification technologies. By discovering the precise molecular target of a drug, researchers can better optimize the drug for a particular disease or condition [2, 3]. Target identification is also important to optimize drug selectivity and reduce its potential side effects [1, 3]. There are several types of biomolecules that can serve as therapeutic targets, including enzymes, cellular receptors, ion channels, DNA, and transcription factors [4–6]. Due to this vast diversity of proteins and other chemicals present in a cell, identifying a specific biological target for a given drug can be extremely difficult [7]. The machine-based and biological experimental-based approaches facilitate the identification of probable drug targets. However, in the context of biochemical approach at the experimental level, one can classify target identification methods into two main strategies, namely affinity-based pull-down methods and label-free techniques. Affinity-based pull-down requires labelling a small molecule with a tag (such as biotin or a fluorescent tag) and then using it to affinity-purify its binding partners from a cell lysate or other protein mixture [8, 9]. In many cases, labelling the tested small molecule can be difficult, limiting the possibilities of using the affinity-based pull-down approach. To avoid this limitation, label-free approaches have been developed to identify the potential targets of small molecules without requiring the molecules to be chemically modified with an affinity tag or a label [10–12]. In addition to the availability of several scientific databases encompassing diverse physical and chemical properties of various ligands and targets, the recent advances in the gen/prote-omics field provide several approaches for drug-target identification both at the machine-based and experimental levels. This paper will review the various techniques and methodologies used for target identification using experimental biological assays, with a particular focus on affinity-based pull-down and label-free approaches. The biological approaches at the cellular level to find new drug targets, like mutagenesis and genetic screening, will also be discussed. Additionally, the strengths and limitations of these approaches will be highlighted briefly. Thus, this review will serve as a valuable resource for researchers and scientists involved in drug discovery and target identification.

## Affinity-based pull-down approach

Affinity purification is a common method for identifying the targets of small molecules. In this method, the tested small molecule is conjugated to an affinity tag such as biotin or immobilized on a resin such as agarose beads. This chemically modified structure is used as a probe molecule that is incubated with cells or cell lysates. After incubation, the bound proteins are purified using the affinity tag. The purified proteins can then be separated and identified using sodium dodecyl sulfate-polyacrylamide gel electrophoresis (SDSPAGE) and mass spectrometry [9, 13–15]. This method provides a powerful and specific tool for studying the interactions between small molecules and proteins, which can be extremely useful in drug discovery and other areas of research. Additionally, it is capable of determining the targets of small molecules with complex structures or tight structure-activity relationships.

Below we discuss recent advances in affinity-based small molecule target identification techniques.

### On-bead affinity matrix approach

**Fig. 1:**
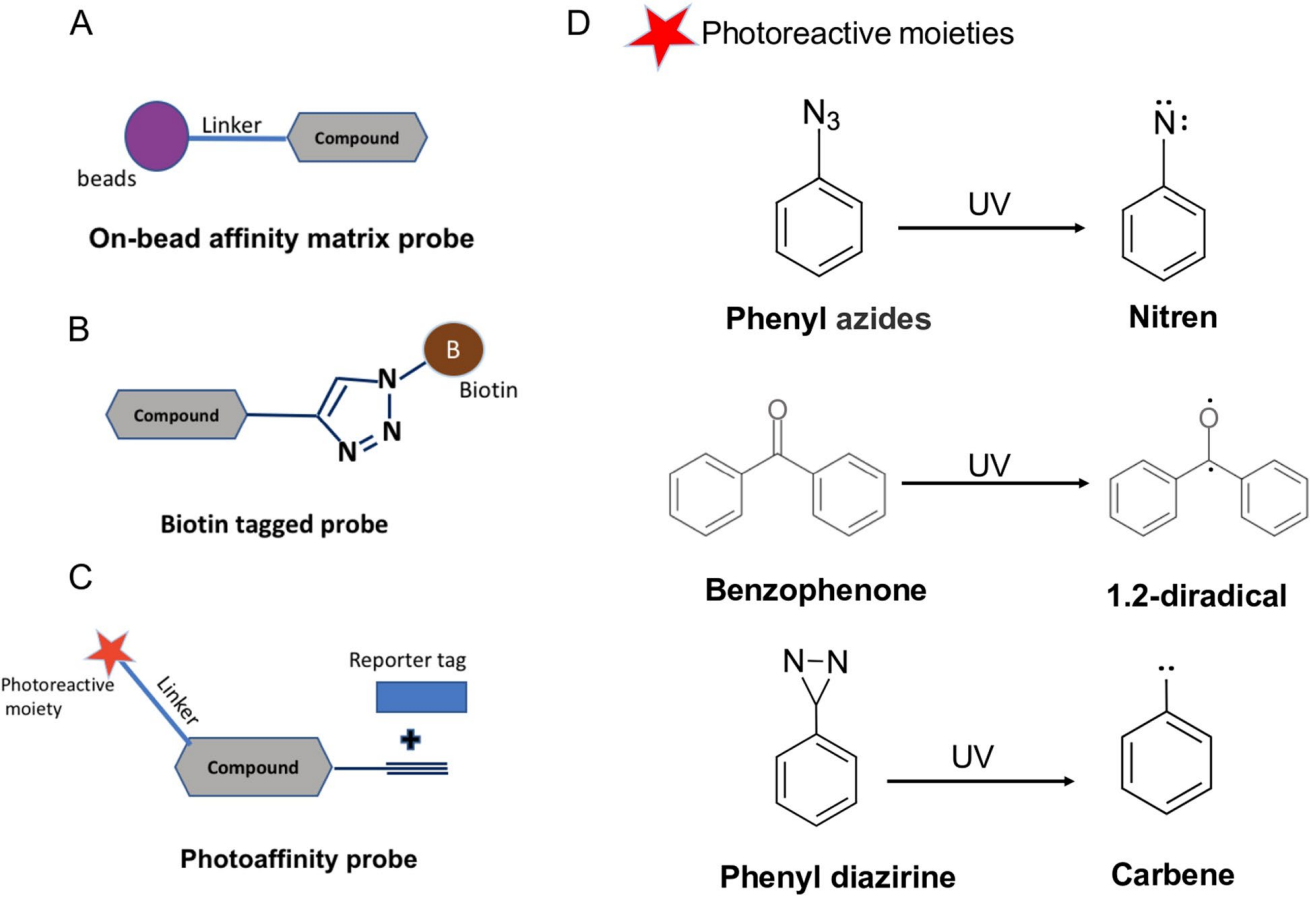
The components of **A** on-bead affinity matrix probe, **B** Biotin-tagged probe, **C** Photoaffinity probe, and **D** Examples of photoreactive moieties and their reactive intermediates

The on-bead affinity matrix approach is a method used to identify the target proteins of biologically active small molecules using an affinity matrix [16]. In this method, a linker, such as polyethylene glycol (PEG), is used to covalently attach a small molecule to a solid support (e.g., agarose beads) at a specific site without changing the small molecule’s original activity of interest (Fig. 1A). The small molecule affinity matrix is then exposed to a cell lysate containing the target protein(s). Any protein that binds to the matrix is eluted and collected for further analysis. Specific target(s) for the tested molecule are then identified using mass spectrometry [17, 18]. As described in Table 1, this method has been adopted successfully by KL001, Aminopurvalanol, Diminutol, BRD0476, and Encephalagen.

**Table 1:** Examples of successful target identification approaches for small molecule

**On-bead affinity matrix approach**			
Compound name	Structure	**Target**	**Ref**
Encephalagen		Ribosomal proteins (S5, S13, S18, ad L28)	[19]
		cryptochrome	[20]
Aminopurvalanol		CDK1	[21, 22]
BRD0476		ubiquitin-specific peptidase 9X (USP9X)	[23]
ssMP C11		F1F0-ATP synthase	[24]
Melanogenin		Prohibitin	[25]
Sulfonyl Amidine		Prohibitin	[26, 27]
TWS119		Glycogen synthase kinase-3 (GSK-3)	[28]
Diminutol		NQO1 (an NADP-dependent oxidoreductase)	[29]
GAPDS		Glyceraldehyde-3-phosphate dehydrogenase	[30]
Quinostatin		Class Ia PI3Ks	[31]
SC1		ERK1- and RasGAP	[32]
QS11		ARFGAP1	[33]
KL001		cryptochrome (CRY)	[20]
**Biotin-tagged approach**			
Withaferin		Type (III) intermediate fila ment (IF) protein, vimentin	[34]
stauprimide		NME2 protein	[35]
Epolactaene		Hsp (heatshock protein) 60	[36]
Chromeceptin		MFP-2	[37, 38]
Myoseverin		Tubulin	[21, 39]
PNRI-299		Redox factor 1 (Ref-1)	[40]
BMS-790052		HCV NS5A	[41]
ICG-001		Cyclic AMP response element-binding protein (CBP)	[42]
**Photoaffinity tagged approach**			
Pladienolide		SF3b	[43]
HUN-7293(2)		Sec61α	[44]
mono-galactosyl-diacylglycerol		TLR4	[45]
kartogenin		Filamin A	[46]
venetoclax		ITPR1GSR RER1 VDAC2 PDIA3	[47]
5′-I fuligocandin B		VCP/p97	[48]
SW208108		SCD	[49]
inflachromene (ICM)		HMGB1, HMGB2	[50]
pyrrolidinone compound 2		fumarate hydratase	[51]
gold N-heterocyclic carbene complex compound (1d)		HSP60 Vimentin NDKA NPM YB-1 PRDX1	[52]
S1-6		Tubulin	[53]
LL-2003		IGF-1R Src	[54]
chamuvarinin derivative compound (1)		mHSP70 ATP5F1	[55]
BIX-01294		*Pf*nPrx NAPL *Pf*HSP110c, etc.	[56]
RYL-634		DHODH	[57]
LBL1		lamin A	[58]
Compound 9im		BCAP31 LPCAT3 POR TM9SF3 SCCPDH CANX	[47]
**Drug affinity responsive target stability (DARTS)**			
5-*epi*-sinuleptolide		actin	[59]
resveratrol		eIF4A	[60]
bithionol		MDH3 GDH1 GND1	[61]
syrosingopine		α-enolase	[62]
Rapamycin		mTOR FKBP12	[10, 60]
nitazoxanide		PAD2	[63]
ellagic acid		ACTN4	[64]
betulinic acid		GRP78	[65]
gephyronic acid		eIF2α	[66]
axitinib		SHPRH	[67]
salinomycin		nucleolin	[68]
cryptotanshinone		FKBP1A	[69]
FK506		FKBP12 calcineurin	[10, 60]
arctigenin		PP2A	[70]
**Stability of Proteins from Rates of Oxidation (SPROX)**			
tamoxifen		YBX-1	[71]
manassantin A		filamin A, EF1α	[72]
**Cellular context thermal shift assays (CETSA)**			
aurone derivative 1a		Class III PI3K (Vps34)	[73]
ferulin C		tubulin	[74]
10,11-dehydrocurvularin		STAT3	[75]
Geranylnaringenin		SH2 domain-containing protein tyrosine phosphatase-2 (SHP-2)	[76]
**Mutagenesis**			
KPT-9274		nicotinamide phosphoribosyltransferase (NAMPT)	[77]
Picolinamide scaffolds		lipid-transfer protein Sec14p, the major phosphatidylinositol-transfer protein (PITP)	[78]
Genetic screening			
BDW568		STING and CES1	[79]

### Biotin-tagged approach

Biotin is a small molecule that is commonly used in affinity-based techniques due to its strong binding affinity to the proteins, avidin and streptavidin. By attaching a biotin tag to a small molecule and then using it to pull out the target protein (Fig. 1B), researchers are able to selectively isolate and identify the target proteins of that small molecule using techniques such as streptavidin-bead affinity purification [80]. Biotin-tagged approaches are widely used in molecular biology and biochemistry to purify and isolate pre-defined or other molecules from complex mixtures. In this method, a biotin molecule is attached to the small molecule of interest through a chemical linkage, and the biotin-tagged small molecule is incubated with a cell lysate or living cells containing the target proteins. SDS-PAGE and mass spectrometry are then used to analyze the target proteins after they are captured on a streptavidin-coated solid support [81, 82]. The biotin-tagged approach was used to successfully identify activator protein 1 (AP-1) as the target protein of PNRI-299 [40], as shown in Table 1.

Using biotin-tagging over other protein isolation techniques has many advantages. This includes its low cost and simple purification and isolation of the target proteins. However, the high affinity of the biotin-streptavidin interaction requires employing harsh conditions in order to break their interaction and release the bound proteins from the resin [83]. One common method to release the bound proteins is to expose the matrix to a denaturing buffer, such as a solution containing SDS and a high temperature in the range of 95–100 °C [84, 85]. This can be considered a disadvantage of using the biotin-tagged approach, as the denaturation conditions may alter the structure or activity of the purified proteins and disrupt the biotin-streptavidin interaction. In some cases, it may be possible to use milder conditions to release the bound proteins, such as lower temperatures or the use of a reducing agent, but these methods may not be effective for all proteins or applications [86]. Additionally, attaching biotin to a small molecule can also affect the cell’s permeability and phenotypic results, which can be a drawback when working with living cells. For example, treating cells with a biotinylated compound can reduce the production of IL-2, Reducing IL-2 production in a short-term cell culture assay may not have immediate harmful effects but can limit the activation and proliferation of immune cells, potentially impacting their response to immune challenges [87, 88]. Due to these limitations, it is important to compare the performance of the biotin-tagged method to that of other affinity purification techniques to decide which method is better for a given application.

### Photoaffinity tagged approach

In the photoaffinity labelling (PAL) approach, a chemical probe covalently binds to its target upon exposure to light. In this method, the probe design involves selecting a photoreactive group (e.g., a linker to connect the photoreactive group to the small molecule) and an affinity tag [89, 90] (Fig. 1C). In this context, the photoreactive moiety is activated through its exposure to light, allowing the probe to form a permanent covalent bond with the target molecule. This is helpful for studying the structure and function of the target molecule because the probe can be used to mark specific sites or regions within the target. There are several types of photoreactive groups that can be used in PAL, including phenylazides, phenyldiazirines, and benzophenones (Fig. 1D). When activated by light, each of these groups make a different kind of reactive intermediate, which has different properties and can be used in different ways [8, 91]. For example, phenylazides form a nitrene upon irradiation with specific wavelengths of light, while phenyldiazirines form a carbene, and benzophenones form a diradical. These highly reactive intermediates can covalently bind to the target protein, enabling researchers to investigate its structure and function in more depth [9]. In addition to the previously mentioned photoreactive groups (i.e., phenylazides, phenyldiazirines, and benzophenones), several other functional groups have been utilized for photoaffinity labelling. These include diazocarbonyls, enones, diazo groups, sulphur radicals, halogenated substrates, diazonium salts, nitrobenzenes, and alkyl derivatives of diazirines and azides, among others. Each of these functionalities possesses its own distinctive properties and can be activated by light to generate reactive intermediates that can covalently bind to the target protein or molecule [92]. The choice of photoreactive group for a PAL experiment will depend on the specific objectives of the study as well as the features of the target protein or molecule. Recently, aryldiazirines have been the most commonly used photoreactive group in PAL. They are particularly favoured due to their good chemical stability and resistance to a wide range of variables, such as temperature, nucleophiles, acidic and basic environments, and oxidizing and reducing agents. The trifluoromethyl derivative of aryldiazirines is particularly popular due to its increased stability and propensity to generate a highly reactive carbene when exposed to specific wavelengths of light. The carbene intermediate can then attach covalently to the target protein or molecule [9, 93].

**Fig. 2:**
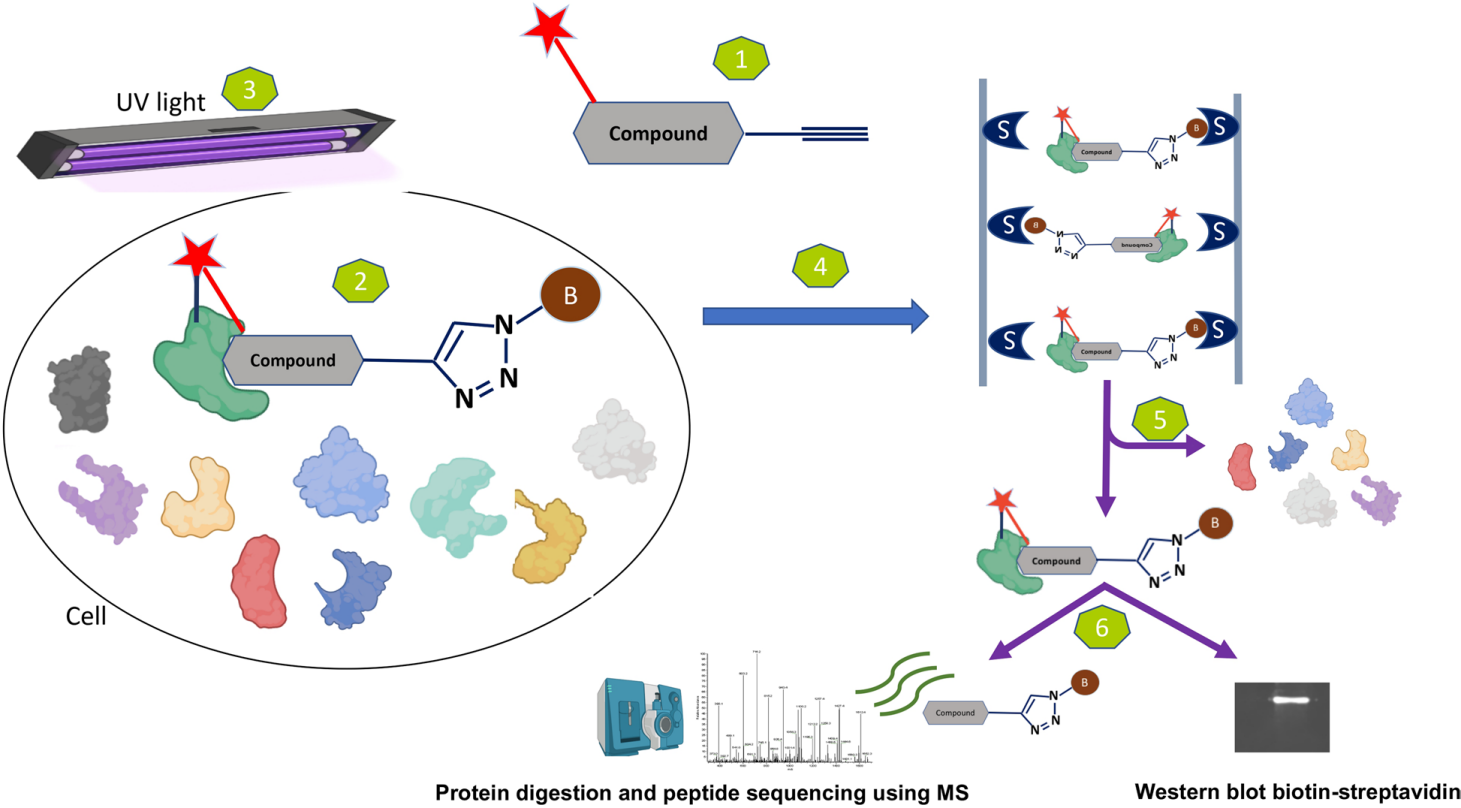
The standard experimental protocol for the photoaffinity method. 1. Synthesizing the photoaffinity probe. 2. Using photoaffinity probes on cells or cell lysates and allowing them to bind to their target. 3. Ultraviolet (UV) light is subsequently employed to activate the covalent crosslinking of the probes with the target proteins in the treated samples (cells or cell lysates). (If the experiment is performed on live cells, the cells are lysed after the UV exposure) proteins. 4.-Using streptavidin, the complex (target + probe) would be extracted. 5. Removing the unbound proteins by washing. 6. The target proteins can be analyzed using SDS-PAGE and identified through protein digestion and mass spectrometry

The PAL approach has several advantages, including a high degree of specificity, allowing for the labelling of the small molecule of interest in a manner that eliminates false positives and improves the precision of the results [90]. Additionally, it could be highly sensitive, enabling the detection of even low levels of protein-ligand interactions. For instance, adding a radiolabel reporter tag offers easy and sensitive detection [94]. Photoaffinity pulldown can also be incorporated into a wide variety of experimental designs [95, 96]. It can also be used to identify proteins that bind to small molecules in numerous cell and tissue types. Furthermore, it enables the identification of protein-ligand interactions, which is useful for understanding the mechanisms of action of small molecules and identifying potential targets for drug development [8, 97]. This approach has been used successfully to identify the target proteins of various small molecules, and various functional handles and photoaffinity linkers have been incorporated to optimize the efficiency of the method. For example, kartogenin that target Filamin A is a compound that promotes the differentiation of multipotent mesenchymal stem cells into chondrocytes [46]. This approach has also been used to identify the target of a small anticancer molecule (LBL1) that was found to bind to the nuclear lamins [58]. The general experimental workflow for the photoaffinity approach is depicted in Fig. 2.

## The limitations and challenges of affinity-based pull-down approaches

Due to the integral roles played by the nature of primary molecule and the linker used to develop the probe, both these factors challenge the utilization of affinity-based pull-down approach. For example, while adopting this technique, it is important to pay attention to the design and synthesis of the modified probes. This step may require frequent testing and evaluation of different probes at different attachment points toward building a structure-activity relationships (SAR) model in order to produce probes that are both effective and specific. Thus, by carefully analyzing the SAR of a given probe arrangement, researchers can identify the structural features that are most vital to its function and use this knowledge to optimize the probe’s design [98]. SAR studies are crucial, which involve the systematic modification of the probe’s structure in order to optimize its binding affinity and specificity for the target protein, as well as its photoreactivity and other characteristics. This can involve making changes to the affinity/specificity unit (small molecule), the linker, the photoreactive moiety, and the identification/reporter tag. There is also the possibility of identifying interactions that are biophysically “real” but not physiologically meaningful, which can be difficult to differentiate without additional validation experiments [8, 10, 98]. The photoreactive group used to label the small molecule may also interfere with its binding to target protein, which may result in false negative results. Another major limitation with affinity-based methods is the need to modify the small molecule with an affinity tag, which can be tedious or impossible for some compounds, and the potential for the affinity tag to alter the biological activity of the small molecule and cause unexpected interactions leading to the identification of off-target binding partners [8, 14, 81, 99, 100]. Lastly, the use of ultraviolet light to trigger the covalent connection of the small molecule to the target protein may be harmful to cells, which may lead to false results [101, 102]. Despite these drawbacks, photoaffinity pulldown remains a valuable tool for studying protein-ligand interactions and can be used to gain insights into the mechanisms of action of small molecules and identify potential drug targets.

On the other hand, the linker or spacer group between the photoaffinity label in a photoaffinity probe and the chemical linker in a biotin-tagged molecule can have a significant impact on its performance. The use of photoaffinity linkers is limited by the possibility of nonspecific binding and random labelling of adjacent proteins. This can occur as a result of the linker’s reactivity, which can cause it to interact with proteins other than the intended target(s) [7, 103]. In addition, if the linker is too short, there is a possibility that the probe will cross-link with itself, which can lead to probe instability and lack of specificity issues. On the other hand, if the linker is too long, the photoreactive group may be too distant from the target protein to grab it efficiently [9]. In general, the ideal linker length is determined by the properties of the probe and the target protein. Therefore, in order to achieve reliable and precise labelling of the target protein, it may be important to carefully select the linker and adjust the conditions for each particular application [13, 104].

The identification component, also known as the reporter tag, is designed to detect the presence of the probe and determine where it is bound to the target. There are numerous forms of available identification tags, including fluorescent dyes, radioisotopes, and particular binding partners like biotin and avidin [94, 105, 106]. With these tags, researchers can use different methods, such as fluorescence microscopy and immunoprecipitation, to find and separate probe-protein adducts. Taken together, due to the challenges associated with the development of SAR, linker and tag to create an efficient molecule, affinity-based pull-down approaches suffer from several disadvantage, including the need for experienced chemists to synthesize the photoaffinity probe, which could be time- and resource-consuming [107].

## Label-free target identification

**Fig. 3:**
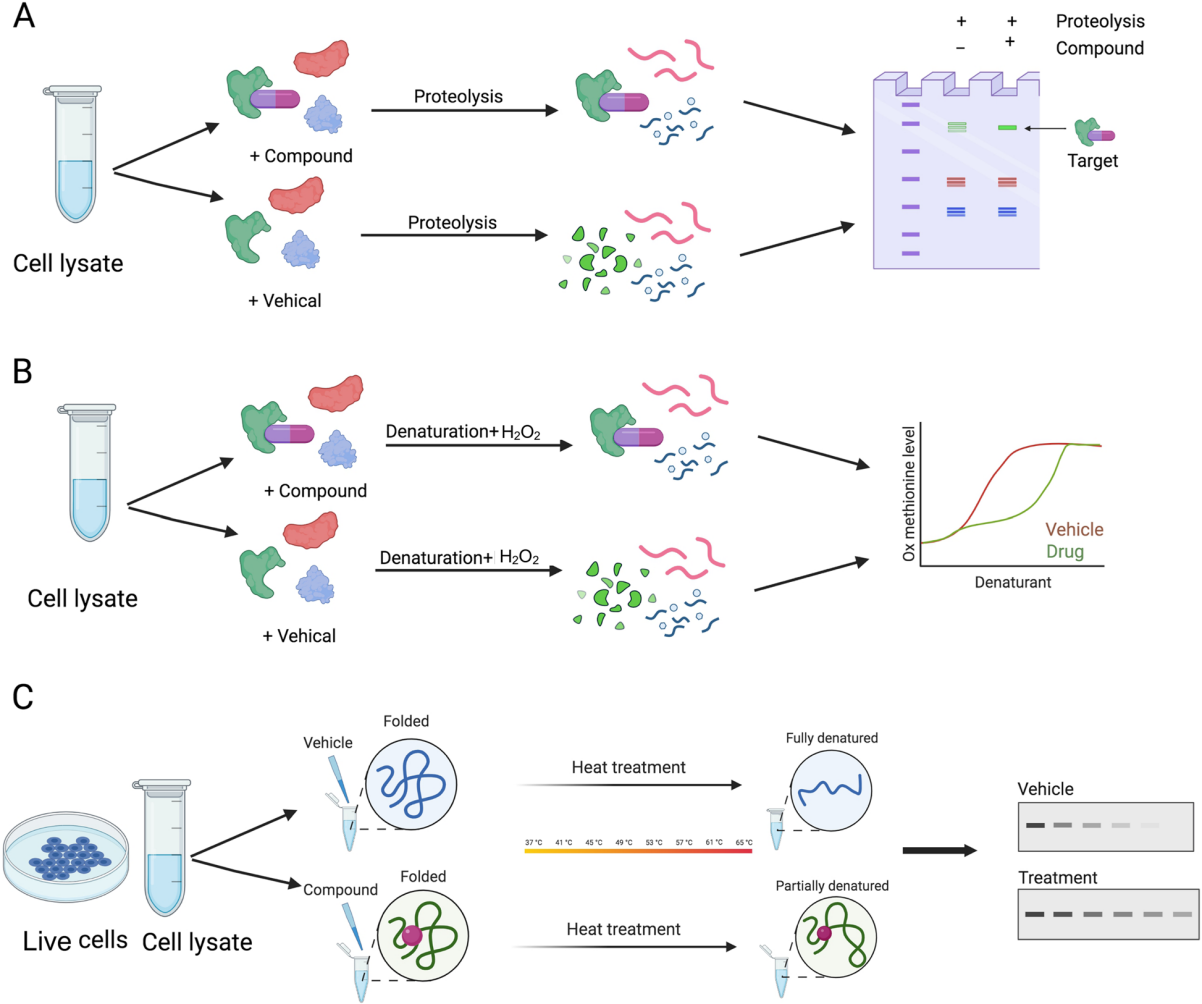
A schematic of label-free target identification approaches. **A** Drug affinity responsive target stability (DARTS), **B** Stability of Proteins from Rates of Oxidation (SPROX), and **C** Cellular thermal shift assay (CETSA).

Label-free approaches utilize the small molecules in their natural state without undergoing any chemical modifications to their structures thus retaining their native confirmation and functional properties of the small molecule. This method is often preferred by the researchers as it does not demand modification or labelling of the primary molecule. Although this approach avoids any potential problems associated with compound labelling, it has few limitations as this label-free molecule can bind to unintended proteins and result in identification of false positive targets [99, 108]. Furthermore, this method is not suitable for proteins that are only expressed at low levels [6, 99]. Below, we will review a few examples of adopting this approach and discuss these limitations in detail (Fig. 3).

### Drug affinity responsive target stability (DARTS)

Drug affinity responsive target stability (DARTS) is a technique developed on the basis that small molecules can bind to and stabilize their target proteins, thereby increasing their resistance to proteolysis (i.e., breakdown through proteases). DARTS utilizes this property to identify the target protein by detecting the binding-induced increase in proteolysis resistance [6, 109, 110]. To perform this, the small molecule is incubated with a cell lysate and then treated with a protease. If the small molecule can bind to its target protein, the protease will be unable to break it down as it increases its stability, resulting in an increase in the amount of protein that remains after treatment. This increase in protein stability can be detected using a technique such as western blotting or mass spectrometry [60]. DARTS is used to identify several protein targets for several small molecules. This technique has proved to be a powerful tool for discovering new small-molecule drugs and for understanding the mechanism of action of these molecules. For example, identifying nucleolin as the binding target of salinomycin, an anticancer stem cell (CSC) small molecule. Table 1 shows examples of the target proteins associated with small molecules using DARTS.

### Stability of proteins from rates of oxidation (SPROX)

The denatured proteins are more susceptible to oxidation than their native counterparts. The Stability of Proteins from Rates of Oxidation (SPROX) method takes advantage of this property by measuring the rates of protein oxidation levels of methionine residues on the protein’s surface in the presence and absence of the small molecule and detecting any changes that may be caused by the small molecule binding to and stabilizing its target protein [6, 111]. In this method, the small molecule is incubated with a cell lysate followed by chemical denaturation treatment and then subjected to an oxidizing agent (H_2_O_2_). The rates of protein oxidation are quantified subsequently using mass spectrometry [112]. The SPROX technique was used to assess the target of tamoxifen, which was found to be Y-box binding protein 1 (YBX1) in MCF-7 cells, where the target was observed to be stabilized by the presence of the small molecule [71]. SPROX is only useful for proteins containing the amino acid methionine. This is due to the fact that SPROX determines the target protein by measuring the level of oxidation of methionine residues in proteins. As a result, SPROX may not be suitable for identifying target proteins that lack methionine residues [112, 113].

It is worth noting that both the SPROX and DARTS approaches are applicable to cell lysates, not living biological systems. As a result, they can only be used to study proteins isolated from cells rather than proteins within cells. This can limit the approaches’ application to specific research questions [112, 114].

### Cellular thermal shift assay (CETSA)

Cellular thermal shift assay (CETSA) was developed according to the concept of ligand-induced thermodynamic stabilization of protein targets. Its increased stability upon ligand binding can be assessed by determining the thermal stability of the protein [115]. Different from SPROX and DARTS techniques, It could be used in live cells and cell lysates. To perform a CETSA, cells or cell lysates are first treated with small molecules or vehicles and then heated. Western blotting is then used to determine if proteins are denatured in a temperature-dependent way and if the melting curves of some proteins that bind to small molecules in samples have shifted. By comparing the thermal stability of the protein with and without the small molecule, it is possible to identify whether a small molecule interacts with a protein and to estimate the association’s affinity [116]. Using a CETSA, it was confirmed that 2′-hydroxy cinnamaldehyde directly binds to STAT3, suppressing STAT3 activity [73]. While this method needs western blotting, which is a limitation of using it due to the availability of antibodies, a number of high-throughput thermal shift approaches for identifying protein targets have been developed, such as MS-CETSA, HCIF-CETSA, and ITDR-MS-CETSA. We will not discuss these techniques here because they have already been thoroughly reviewed in earlier reviews [6, 117, 118].

## Mutagenesis

Mutagenesis is a promising genetic tool to identify drug targets, which involves the manipulation of the expression or function of genes or proteins by altering a specific sequence of DNA or amino acids and observing the resultant effect of this mutation on the drug’s response [119]. The messenger RNA (mRNA) knockdown, site-directed and random genome mutagenesis are the various ways of genetic screening approaches used in drug-target identification. Among them, clustered regularly interspaced short palindromic repeats (CRISPR)-Cas9 mutagenesis has been increasingly gaining popularity as a way to generate a pool of genes which can be used to identify both the cellular target protein and the molecular interaction site of a small-molecule drug candidate. Recently, a CRISPR-tiling–mediated mutagenesis has been reported to be an ideal target fishing approach to identify nicotinamide phosphoribosyltransferase (NAMPT) as the primary molecular target of KPT-9274, an anticancer agent in clinical investigation [77, 120]. This method involves the systematic designing of large single-guide RNA (sgRNA) gene tiling libraries to target and mutate specific genes of known anticancer drugs and developing constructs of lentiviral libraries which can be transduced to generate mutagenized cells. These cells could be tested for their functionality with the related known drug-target pairs to identify the cellular target(s) of a small molecule. Notably, a classic study by Pries et al. identified the fungal lipid-transfer protein Sec14p, the major phosphatidylinositol-transfer protein (PITP) in S. cerevisiae, as the primary target of benzamide and picolinamide scaffolds exhibiting antifungal activities [78].

Mutagenesis offers the advantage of revealing a drug’s direct and indirect interactions with its target and other cellular components. Additionally, it can facilitate the optimization of lead compounds by modifying their structure or activity. Moreover, a large diversity of genetic variants generated by mutagenesis can be screened not only to detect drug sensitivity but also to understand drug resistance. Mutagenesis can help uncover primary and secondary targets and pathways involved in the drug’s mode of action [108]. On the other hand, the disadvantages associated with this method involve being time-consuming, labor-intensive, and low in efficiency, especially for complex genomes and phenotypes resulting, which can introduce unwanted or off-target effects that can confound the interpretation of the results. Mutagenesis can be inaccurate or incomplete, resulting in false positives or negatives. Mutagenesis can be complicated by the presence of multiple targets, redundant targets, or compensatory mechanisms that can mask the effect of a single mutation [121].

## Genetic screening

Genetic screening is another unique, unbiased method for cellular drug target identification [119, 122]. In this method, a knockout library of the selective target(s) of interest was designed (utilizing RNA interference (RNAi) or CRISPR-Cas9) and screened for loss-of-function of the probable drug target(s). In a pivotal study, screening the siRNA library of genes (related to kinases and cellular proteins) helped to identify both the known and novel target genes modulated by TRAIL, resulting in the induction of apoptosis [123]. In a recent seminal study, a CRISPR-based target identification platform with an inducible suicide gene expression system was utilized to positively enrich the cells bearing the knocked-out target that was identified by sequencing of gRNA sequences and loss of function [79]. Using this platform, the authors confirmed STING and CES1 as the primary target and a key metabolizing enzyme, respectively, of a small molecule IFN-I activator, BDW568, in cells. In contrast to the conventional CRISPR-based target screening relying on the antiproliferative effects of the drugs, this smart method can be adapted to any drug with non-proliferative activity. Using a library of clinically relevant kinase inhibitors and utilizing nearly six thousand drug-gene pairs, NOTCH1 and its downstream signaling pathway are identified to be involved in drug resistance in breast cancer cells [124]. This method suffers from some of the challenges associated with mutagenesis.

## Conclusion

Protein target identification research is an important part of the drug discovery process and requires a significant investment of time and resources. Through careful consideration of the main advantages and limitations of affinity-based pull-down, label-free, mutagenesis and genetic screening approaches, researchers are able to make well-informed decisions when selecting a target identification strategy for drug discovery. Choosing the most appropriate approach depends on the specific requirements of the research project.

## Data Availability

All data generated or analyzed during this study are included in this published article.
